# Childhood growth and neurocognition are associated with distinct sets of metabolites

**DOI:** 10.1016/j.ebiom.2019.05.043

**Published:** 2019-05-25

**Authors:** G. Brett Moreau, Girija Ramakrishnan, Heather L. Cook, Todd E. Fox, Uma Nayak, Jennie Z. Ma, E. Ross Colgate, Beth D. Kirkpatrick, Rashidul Haque, William A. Petri

**Affiliations:** a Division of Infectious Diseases and International Health, University of Virginia, Charlottesville, VA, USA; b Department of Statistics, University of Virginia, Charlottesville, VA, USA; c Department of Pharmacology, University of Virginia, Charlottesville, VA, USA; d Center for Public Health Genomics, University of Virginia, Charlottesville, VA, USA; e Department of Public Health Sciences, University of Virginia, Charlottesville, VA, USA; f Vaccine Testing Center, Department of Microbiology and Molecular Genetics, Larner College of Medicine, University of Vermont, Burlington, VT, USA; g International Centre for Diarrheal Disease Research, Dhaka, Bangladesh

**Keywords:** Metabolomics, Stunting, Neurocognition, Childhood, Phosphatidylcholine, Sphingomyelin

## Abstract

**Background:**

Undernutrition is a serious global problem that contributes to increased child morbidity and mortality, impaired neurocognitive development, and decreased educational and economic attainment. Current interventions are only marginally effective, and identification of associated metabolic pathways can offer new strategies for intervention.

**Methods:**

Plasma samples were collected at 9 and 36 months from a subset of the PROVIDE child cohort (*n* = 130). Targeted metabolomics was performed on bile acids, acylcarnitines, amino acids, phosphatidylcholines, and sphingomyelins. Metabolic associations with linear growth and neurocognitive outcomes at four years were evaluated using correlation and penalized-linear regression analysis as well as conditional random forest modeling.

**Findings:**

Different metabolites were associated with growth and neurocognitive outcomes. Improved growth outcomes were associated with higher concentrations of hydroxy-sphingomyelin and essential amino acids and lower levels of acylcarnitines and bile acid conjugation. Neurocognitive scores were largely associated with phosphatidylcholine species and early metabolic indicators of inflammation. All metabolites identified explain ~45% of growth and neurocognitive variation.

**Interpretation:**

Growth outcomes were predominantly associated with metabolites measured early in life (9 months), many of which were biomarkers of insufficient diet, environmental enteric dysfunction, and microbiome disruption. Hydroxy-sphingomyelin was a significant predictor of improved growth. Neurocognitive outcome was predominantly associated with 36 month phosphatidylcholines and inflammatory metabolites, which may serve as important biomarkers of optimal neurodevelopment. The distinct sets of metabolites associated with growth and neurocognition suggest that intervention may require targeted approaches towards distinct metabolic pathways.

**Fund:**

Bill & Melinda Gates Foundation (OP1173478); National Institutes of Health (AI043596, CA044579).

**Research in context**

**Evidence before this study**

Undernutrition is a global health problem with long-term consequences on mortality, neurodevelopment, and economic achievement. Previous studies have found that nutritional interventions have only a modest effect on growth recovery and optimal neurodevelopment. Multiple causes have been identified for stunting, including insufficient diet and environmental enteric dysfunction, a disease of malabsorption and chronic intestinal inflammation. Subsequent metabolomic studies have identified metabolic pathways associated with disease, but these studies have primarily focused on growth with only limited investigation into the role of metabolic pathways on neurocognition.

**Added value of this study**

The current study utilized targeted metabolomics to compare plasma metabolites to outcomes of four year growth and neurodevelopment in an undernourished child cohort. Both penalized linear regression and conditional random forest methods were used to select predictors for each outcome. Distinct metabolic pathways were shown to influence growth and neurocognition. Plasma hydroxy-sphingomyelin species were identified as significant predictors of linear growth at four years as well predictors of neurocognitive development to a lesser extent. Neurocognitive outcome at four years was predominantly associated with phosphatidylcholine species from three year plasma.

**Implications of all available evidence**

The differences between metabolites identified with growth and neurocognitive outcomes suggest that separate, more targeted interventions may be required for these outcomes. Growth outcomes were associated with early time point metabolites, particularly metabolic markers associated with poor diet and environmental enteric dysfunction. Hydroxy-sphingomyelin species are also potential biomarkers of poor growth. Neurocognition was predominantly associated with phosphatidylcholines measured later in development and metabolic indicators of inflammation, implying that environmental stressors or current health status may be the most pressing targets for intervention into this outcome.

Alt-text: Unlabelled Box

## 1 Introduction

Undernutrition is a severe global health problem, contributing to 45% of deaths in children under the age of five [1]. The most striking manifestation of undernutrition is linear growth stunting: a UNICEF-WHO-World Bank Joint study estimates that 165 million children under the age of five are stunted, with stunting most prevalent in Africa and Asia [2]. While stunting is defined as a height-for-age Z (HAZ) score of less than −2.0 (two standard deviations below the population median), a sizeable number of children that do not meet this threshold still fail to reach proper growth milestones. In addition to increased mortality, stunting in early life can have lifelong consequences, leading to impaired neurocognitive development [3,4] and decreased educational and economic attainment [[5], [6], [7]]. This chronic, lifelong stunting can be passed from mother to neonate before birth [8], reflecting a cyclical “stunting syndrome” that affects multiple generations [9]. Several drivers of stunting have been identified, including insufficient diet [10], frequent infections [11], and environmental enteric dysfunction (EED), a condition of chronic, low level intestinal inflammation associated with poor sanitation [12,13]. Infections and EED have also been linked to poor neurodevelopment [14], although interactions between inflammation, stunting, and neurodevelopment make it difficult to tease apart the contributions of each to disease.

While several causative factors for stunting and impaired neurodevelopment have been identified, current known interventions have been ineffective at alleviating disease. Post-natal nutrient supplementation trials have shown only modest efficacy at correcting linear growth defects [15,16] and proved ineffective at reversing impaired neurodevelopment [5,17] in early life. Previous estimates predict that applying all known interventions at 99% efficiency would result in only a 33% decrease in stunting and a 25% decrease in childhood mortality by three years of life [18]. This finding suggests that there is still a gap in knowledge with regard to what biological pathways can be targeted to intervene in childhood stunting. Several studies have identified metabolic differences associated with poor linear growth or EED [[19], [20], [21], [22], [23]], but these results must be replicated in additional populations to control for cultural or dietary differences. In addition, there have been limited studies investigating how metabolic differences may influence neurocognition within the first years of life, making this a critical area of investigation.

In the current study, targeted metabolomics was performed on plasma samples collected from a 130 child subset of the PROVIDE Bangladeshi growth cohort. Previous analyses of the data collected in this cohort have found associations between maternal and EED biomarkers each with stunting and vaccine failure [24] as well as associations between inflammation and neurocognitive outcomes [25,26]. The random forest method was also used to assess the importance of these factors to predict linear growth and neurodevelopment at two years, determining that maternal and birth anthropometry were the greatest predictors of linear growth outcomes, while inflammatory biomarkers were the most commonly associated predictors with neurodevelopment [27]. However, the underlying metabolic pathways that influence these outcomes have not been investigated.

The objective of this study was to identify metabolic associations with growth and neurocognitive outcomes. Several classes of metabolites were selected for analysis based on previous associations with EED and growth stunting, including bile acids [28], amino acids [20,23], and acylcarnitines [22]. Plasma samples were measured at 9 and 36 months, allowing for identification of the timing of when specific metabolic pathways are important. The growth and neurocognitive outcomes analyzed include height-for-age Z (HAZ) score as a measure of growth stunting, ∆HAZ (the difference between HAZ at enrollment and at four years) as a measure of the trajectory of stunting, and full-scale intelligence quotient (IQ) scores derived from the Wechsler Preschool and Primary Scale of Intelligence (WPPSI-III) as a measure of neurocognitive development. Identifying the relationships between metabolites and these outcomes will elucidate pathways associated with growth and neurocognitive impairment that may serve as biomarkers of disease or targets for potential interventions.

## 2 Materials and methods

### 2.1 Study population

The Performance of Rotavirus and Oral Polio Vaccines in Developing Countries (PROVIDE) study is a longitudinal study designed to examine oral vaccine efficacy in an undernourished population in Bangladesh. The study design and population of this study has been previously described [29]. Briefly, 700 children were enrolled within the first week of life and followed up to two years while collecting anthropometric growth data as well as stool and serum samples. For the current study, a smaller subset (130) of children from the PROVIDE cohort was followed further beyond two years with anthropometry and neurocognitive assessment using the Wechsler Preschool and Primary Scale of Intelligence, Third Edition (WPPSI-III) at 4 years of age. HAZ and ΔHAZ growth measurements were taken throughout the first two years of life and then at 6 month intervals up to five years of age. On average, HAZ declined in both the full PROVIDE cohort and the 130 child subset to two years of age, at which point HAZ plateaued.

Neurocognitive assessment with the WPPSI-III in Bangladesh has previously been described [30]. Briefly, the WPPSI-III was culturally adapted for use with Bangladeshi children and assessments were administered at the nearest health clinic. Local staff were trained to administer the test to children and adequate inter-observer and test-retest reliability were achieved. The WPPSI-III assesses performance IQ (using Block Design, Matrix Reasoning, and Picture Completion subtests) and verbal IQ (using Information, Vocabulary, and Comprehension subtests), and these scaled scores are summed to calculate the full-scale IQ score.

### 2.2 Sample preparation

Plasma samples were collected within seven days of the exact date for the 9 month and 36 month time points for each child. Samples were stored at 4 °C until they were brought to the International Centre for Diarrheal Disease Research, Bangladesh (icddr,b), where they were frozen at −80 °C within four hours of collection. Plasma samples taken at 9 and 36 months of life were shipped from icddr,b to the University of Virginia, where they were stored at −80 °C until use. Before use in metabolomics assays, plasma was distributed into aliquots for use in multiple metabolomic assays.

### 2.3 Metabolite measurement and data validation

Plasma metabolites were analyzed using the AbsoluteIDQ p180 and Bile Acids kits (Biocrates Life Sciences AG, Innsbruck, Austria). The p180 kit is a targeted metabolomics approach for identifying 185 metabolites, including acylcarnitines, amino acids, biogenic amines, phosphatidylcholines, and sphingomyelins, while the Bile Acids kit detects 20 bile acids. Before use in metabolomics assays, plasma samples were thawed, briefly vortexed, then spun at 2800 x*g* for five minutes. Supernatant (10 μl) was then added to filter plates and samples were processed according to manufacturer's protocol. All reagents used during sample preparation were UHPLC-MS grade. Samples were analyzed by LC-MS/MS on a Waters (Milford, MA) I-Class Acquity chromatography system in-line with a Waters TQ-S mass spectrometer. Compounds were analyzed using TargetLynx XS software with the results subsequently imported into MetIDQ software (Biocrates) for quality control (QC) validation.

Raw metabolite concentrations (μM) were normalized using identical QC samples throughout all plates to control for plate to plate variation. QC samples were proprietary samples spiked with metabolites measured during targeted metabolomics. Normalized metabolite concentrations were exported for further analysis and screened for inclusion in the data set based on two criteria. First, metabolites must be within the valid range in at least 66% of quality control samples run on a plate to be included in analysis. Second, metabolites must be above the limit of detection in at least 60% of all measurements to be included in the valid data set. The QC screen eliminated 4 metabolites from analysis, while the limit of detection screen eliminated 84 metabolites from analysis. Data set validation was performed using R software version 3.4.3 (https://cran.r-project.org).

### 2.4 Metabolite nomenclature

All metabolites are described using the nomenclature described by Biocrates. Acylcarnitines in this kit are marked by their carbon chain length (C0-C18:2). All metabolites marked with PC are phosphatidylcholine species. The aa or ae designations indicate whether the fatty acid chain is bound by an ester (aa) or ether (ae) bond. The numbers (x:y) represent the total carbon chain length (x) followed by the number of double bonds (y) in the fatty acid chains. All metabolites marked with SM are sphingomyelin species, with SM[OH] representing hydroxy-sphingomyelin species. All metabolites in a class were summed to provide a “total” measurement which was also compared to outcomes of interest. In addition, the ratio of conjugated to unconjugated bile acids and the ratio of kynurenine to tryptophan were also calculated from valid individual measurements taken by the kit.

### 2.5 Statistical analysis

Initial screening of data sets was performed using Pearson correlation between all valid metabolites and the three outcomes of interest: HAZ, ∆HAZ, and WPPSI-III full-scale IQ score at four years. Associations were defined as a Pearson correlation coefficient of *r* > 0.2 or *r* < −0.2. All statistical analyses were performed using R software version 3.4.3 (https://cran.r-project.org). Pearson correlations were calculated using the ‘Hmisc’ package (version 4.1–1) and further adjusted using the False Discovery Rate (FDR) method to account for multiple comparisons for all metabolites measured at a given measurement time. SCAD-penalized linear regression was performed using the ‘grpreg’ package (version 3.2–0). Conditional random forest was performed using the ‘party’ package (version 1.3–1). Figures were generated using Prism 8 software (Graphpad) and R, using the ‘tidyverse’ (version 1.2.1) and ‘ggrepel’ (version 0.8.0) functions to generate volcano plots.

### 2.6 Ethics statement

The PROVIDE study was approved by the Research Review and Ethics Review Committees at The International Centre for Diarrheal Disease Research, Bangladesh and by the Institutional Review Boards at the University of Virginia, the University of Vermont, and Harvard University. Informed consent was obtained from parents before child participation in the study, and all data was anonymized prior to analysis.

## 3 Results

### 3.1 Study population characteristics

**Fig. 1:**
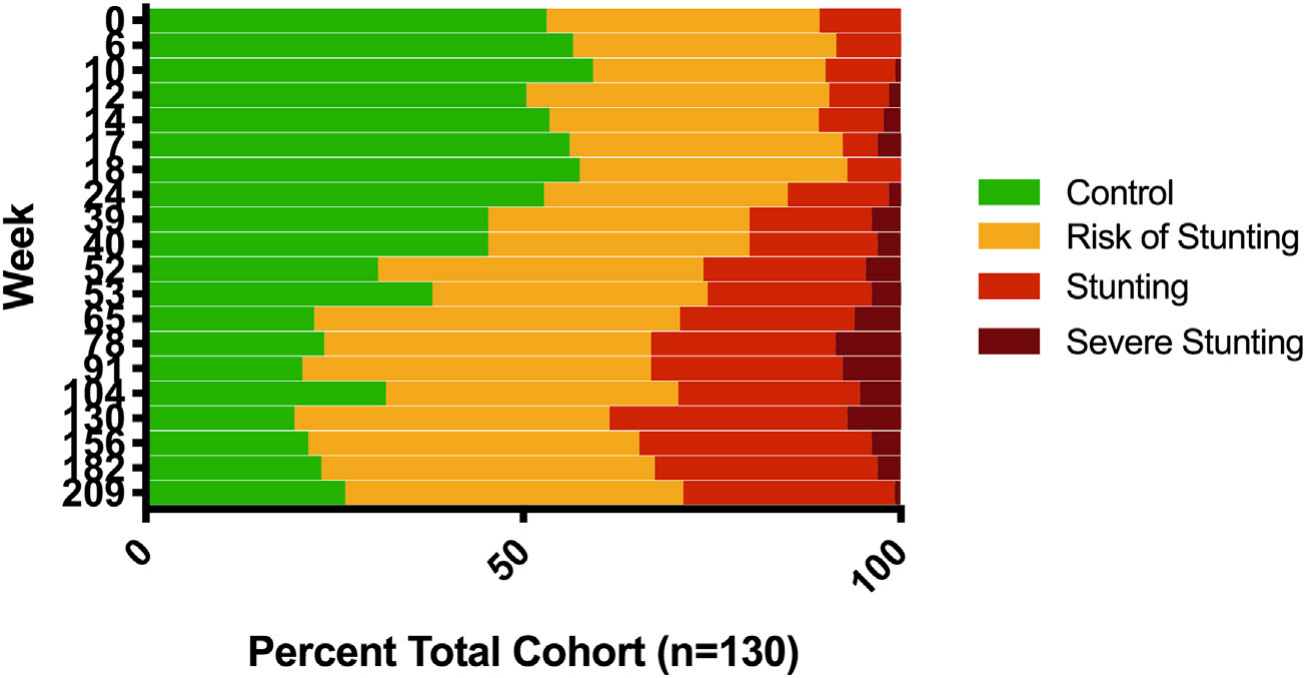
Progression of stunting in the PROVIDE neurocognitive subset: HAZ measurements were taken from enrollment through four years of age. HAZ measurements were categorized as control (HAZ > −1.0), risk of stunting (HAZ −1.0 to −2.0), stunting (HAZ −2.0 to −3.0) or severe stunting (HAZ < −3.0). The week of each HAZ measurement is indicated on the y-axis, with total percentage for each category indicated on the x-axis.

Demographic characteristics for both the full PROVIDE cohort and the 130 children analyzed in the current study are presented in Table 1. The smaller study cohort was representative of the full PROVIDE cohort in regards to age, sex, maternal education, monthly income, and enrollment HAZ, while there were significant differences between HAZ and ∆HAZ at two years as well as the number of days of exclusive breastfeeding. The significant differences in growth between PROVIDE and the smaller subset are likely due to changes in the population as a whole over time, as the subset was composed of children enrolled in the last six months of the original study and both HAZ and ∆HAZ values increased over the course of PROVIDE enrollment (Supplemental Fig. 1). Comparisons between the full cohort and the study cohort are primarily taken at two years, as data was not collected on the larger PROVIDE cohort after this point. Progression of linear growth stunting in the study cohort over the first four years of life is illustrated in Fig. 1. The prevalence of stunting in this subset was 10.8% at enrollment and steadily increased to a rate of 29.6% at two years, after which it remained fairly stable. In addition to the portion of this cohort that was stunted, a substantial portion was at risk for stunting, as defined by −1 > HAZ > −2. Because of the large number of individuals at risk of stunting, data was not categorically separated by stunting status but instead analyzed using HAZ as a continuous variable. The average ∆HAZ scores at two years was calculated as −0.49 ± 0.90, with lower ∆HAZ scores representing worse growth outcome. Neurocognitive development was assessed at four years of age for this study cohort, with average WPPSI-III scores of 84.6 ± 6.4, 81.8 ± 8.6, and 80.7 ± 8.0 for verbal IQ, performance IQ, and full-scale IQ, respectively.

**Table 1:** Descriptive characteristics of total PROVIDE cohort and study subset.

Characteristic	Total (N=700)	Subset (N=130)	P Value^a^
Male sex (%)	368 (52.6%)	72 (55.4%)	0.4773
No maternal education (%)	202 (28.3%)	43 (33.1%)	0.9384
Monthly income, BDT	12,762 ± 9410	12,775 ± 8981	0.9869
HAZ, Enrollment	−0.90 ± 0.89	−0.94 ± 0.81	0.5673
HAZ, Two Years	−1.61 ± 1.04	−1.43 ± 0.95	**0.0277**
Delta HAZ, Two Years	−0.70 ± 1.02	−0.49 ± 0.90	**0.007**
WIS-C Score, Four Years			
Verbal IQ	–	84.58 ± 6.38	–
Performance IQ	–	81.80 ± 8.63	–
Full Scale IQ	–	80.69 ± 7.96	–
Days Exclusive Breast Feeding	110 ± 62	125 ± 59	**0.0023**
Diarrheal Days, Two Years	30.3 ± 24.1	33.9 ± 26.7	0.0559

a Calculated from two-tailed Student's *t*-test comparing the metabolic subset with total PROVIDE (excluding the metabolic subset, *n* = 570). Bolded values are significant (P < 0.05) in statistical tests.

### 3.2 Associations between plasma metabolites and four year outcomes

Targeted metabolite analysis was carried out to quantify metabolites from 9 and 36 month plasma samples. Metabolites were initially screened for associations with four-year growth and neurocognitive outcomes by correlation analysis, with association defined as a Pearson's correlation coefficient of *r* > 0.2 or *r* < −0.2. The characteristics of associated metabolites with regard to age of collection and class of metabolite are presented in Table 2. Overall, 34 metabolites were associated with HAZ, 37 with ∆HAZ, and 34 with full-scale IQ. A majority of metabolites associated with linear growth outcomes (HAZ and ∆HAZ) were from 9 month plasma samples, while metabolites associated with full-scale IQ score were disproportionately from 36 month plasma samples. All associated metabolites were significantly correlated before adjustment for multiple comparisons. However, due to the large number of metabolites analyzed, only a limited number of metabolites met a false discovery rate (FDR) threshold for significance. Correlations with and without FDR adjustment for all metabolites are reported in Supplemental Table 1.

**Fig. 2:**
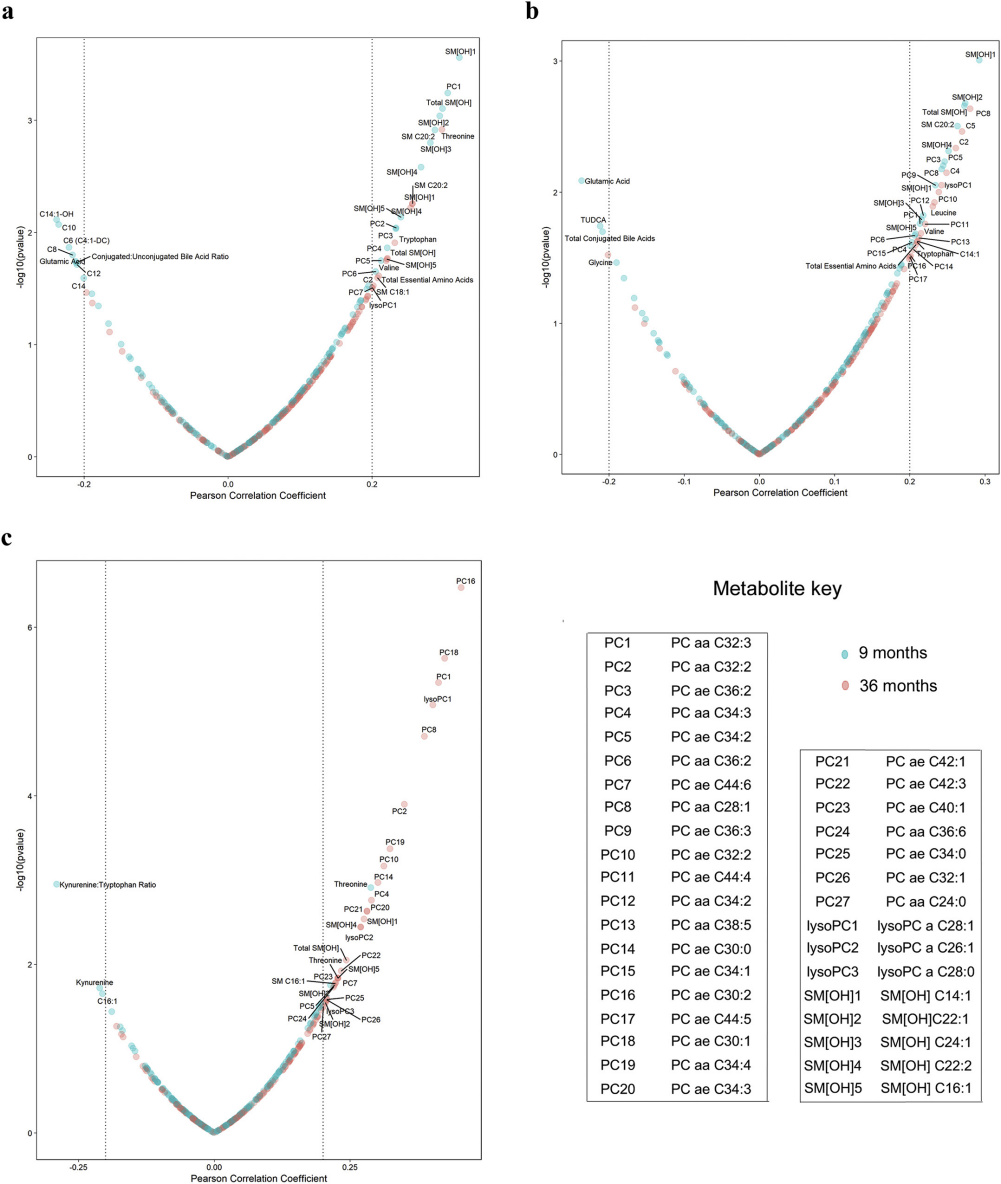
Associations between plasma metabolites and four year outcomes: Volcano plots of associations between plasma metabolites and (a) HAZ, (b) ∆HAZ, and (c) full-scale IQ score. Pearson correlation coefficient is given on the x-axis, while -log10(pvalue) is given on the y-axis. Dotted vertical lines indicate Pearson correlation coefficient *r* > 0.2 and *r* < −0.2, the cutoffs used for association with each outcome. All labeled metabolites met the cutoff for association. The colour of each data point indicates the timepoint at which it was collected, with nine month metabolites shown in blue and 36 month metabolites shown in red. (For interpretation of the references to colour in this figure legend, the reader is referred to the web version of this article.)

The relationship between individual metabolites and four year outcomes are shown in Fig. 2. The 34 metabolites associated with HAZ were primarily hydroxy-sphingomyelin (SM[OH]) and phosphatidylcholine (PC) species, which were positively associated (Fig. 2A). SM[OH] species were the most prevalent and most strongly associated metabolites with growth, as 80% (8/10) of SM[OH] metabolites measured were positively associated with HAZ. Total SM[OH], which represents the sum of five individual species measurements (SM[OH] C14:1, SM[OH] C16:1, SM[OH] C22:1, SM[OH] C22:2, SM[OH] C24:1), was associated with HAZ at both 9 and 36 months. Essential amino acids at 36 months were also positively associated with HAZ, driven primarily by the amino acids threonine, tryptophan, and valine. Most metabolites were positively associated with HAZ, but negative associations were also observed with several metabolites. While C2 acylcarnitine was positively associated with HAZ, medium to long chain acylcarnitines (C8, C10, C12, C14, C14:1-OH) were negatively associated. In addition to acylcarnitines, poor growth was also associated with a higher ratio of conjugated to unconjugated bile acids as well as the amino acid glutamic acid in 9 month plasma.

**Table 2:** Characteristics of metabolites associated with four year outcomes.

Characteristic	HAZ	ΔHAZ	Full-scale IQ
Total metabolites associated	34	37	34
Metabolites by plasma sample date			
9 Month	21/34 (61.8%)	19/37 (51.4%)	5/34 (14.7%)
36 Month	13/34 (38.2%)	18/37 (48.6%)	29/34 (85.3%)
Metabolites by classification			
Acylcarnitine (C)	7/34 (20.6%)	4/37 (10.8%)	1/34 (2.9%)
Amino acid/biogenic amines	5/34 (14.7%)	6/37 (16.2%)	4/34 (11.8%)
Bile acid	1/34 (2.9%)	2/37 (5.4%)	0/34 (0.0%)
Phosphatidylcholine (PC)	8/34 (23.5%)	17/37 (45.9%)	22/34 (64.7%)
Sphingomyelin (SM)	13/34 (38.2%)	8/37 (21.6%)	7/34 (20.6%)

Many of the metabolites associated with HAZ were also associated with ∆HAZ. The predominant metabolites associated with ∆HAZ were once again SM[OH] and PCs, although fewer SM[OH] species were associated with ∆HAZ compared to HAZ (Fig. 2B). Essential amino acids at 36 months were also positively associated with ∆HAZ, here driven by leucine, tryptophan, and valine, while glutamic acid was again negatively associated with growth. Bile acid conjugation was once again associated with poor growth, as total bile acid conjugation was negatively associated with ∆HAZ. Medium-to-long chain acylcarnitines, which had been associated with poor HAZ at 4 years, were not associated with ∆HAZ. Rather, short chain acylcarnitines from 36-month plasma were associated with positive ∆HAZ outcomes, including C2, C4, and C5.

The set of metabolites associated with full-scale IQ score was fairly different from those associated with linear growth outcomes. While growth was associated with metabolites from all classes, neurocognitive outcomes were predominantly associated with PC species from 36 month plasma, constituting the eight most highly associated metabolites and 64.7% of all metabolites associated with full-scale IQ score. Several SM[OH] species were also associated with full-scale IQ, and these metabolites were also mostly derived from 36-month plasma. Bile acids, acylcarnitines, and amino acids were generally not associated with neurocognitive outcome. Threonine, the only amino acid associated with full-scale IQ, was positively associated with neurocognitive scoring at both 9 and 36 months. The only metabolites to be negatively associated with neurocognitive outcome were kynurenine and the kynurenine:tryptophan ratio.

### 3.3 SCAD-penalized linear regression

While a number of metabolic associations were identified, selecting which variables are most important or predictive for outcomes of interest can be difficult when working with large data sets. Penalized linear regression analysis represents an accurate method of identifying predictors from data with a large number of variables or with variables that are highly correlated [31], making it ideal for variable selection from metabolic data sets. Therefore, linear regression with a SCAD penalty was used to identify predictors from metabolites associated with four year outcomes. Before analysis, all metabolites associated within a given outcome were tested for correlations among themselves and single representative metabolites were chosen from any group with Pearson correlation coefficients of *r* > 0.6. Penalized linear regression was then used on this narrowed down data set to select predictors of four year outcomes.

The metabolites chosen by SCAD as well as the direction of the association between metabolites and outcomes are listed in Table 3. Overall, metabolites selected by SCAD were representative of the metabolites associated with four year outcomes as a whole. Many of the predictors for HAZ were acylcarnitines, with C2 from 36 month plasma being positively associated and C10 and C14:1-OH from 9 month plasma being negatively associated. HAZ was also associated with the conjugated:unconjugated bile acid ratio and total SM[OH] at 9 months as well as threonine at 36 months. Many of the same metabolites or metabolic pathways predictive of HAZ were also selected by SCAD for ∆HAZ. Total SM[OH] at 9 months and C2 at 36 months, which were positive predictors of HAZ, were also positive predictors of ∆HAZ. While the ratio of conjugated:unconjugated bile acids was negatively associated with HAZ, total conjugated bile acids and taurine-conjugated UDCA (TUDCA) in particular were selected for ∆HAZ. There were several metabolites that were distinctly selected as ∆HAZ predictors, including several amino acids (leucine, glycine) and 6 PC species, while only one PC species was predictive of HAZ. Metabolites associated with full-scale IQ were predominantly 36 month PC species (PC aa C32:3, PC ae C30:2, and PC ae C34:3), along with threonine from both 9 and 36 months and 9 month kynurenine. While threonine from 36 month plasma was also selected by SCAD for HAZ, full-scale IQ predictors were largely distinct from those of linear growth.

**Table 3:** Metabolomic predictors selected by SCAD-penalized linear regression for four year outcomes.

Outcome	Metabolite	Time of measurement	Direction of association
HAZ at 4 years	Conjugated:unconjugated bile acid ratio	9 months	(−)
	C10	9 months	(−)
	C14:1-OH	9 months	(−)
	Total SM[OH]	9 months	(+)
	C2	36 months	(+)
	Threonine	36 months	(+)
	PC ae C44:6	36 months	(+)

∆HAZ from enrollment to 4 years	TUDCA	9 months	(−)
	Total conjugated bile acids	9 months	(−)
	Glutamic Acid	9 months	(−)
	PC aa C28:1	9 months	(+)
	PC aa C34:2	9 months	(+)
	PC aa C34:3	9 months	(−)
	Total SM[OH]	9 months	(+)
	C2	36 months	(+)
	Glycine	36 months	(−)
	Leucine	36 months	(+)
	PC aa C28:1	36 months	(+)
	PC aa C38:5	36 months	(+)
	PC ae C44:4	36 months	(+)

WPPSI-III full scale IQ score at 4 Years	Threonine	9 months	(+)
	Kynurenine	9 months	(−)
	Threonine	36 months	(+)
	PC aa C32:3	36 months	(+)
	PC ae C30:2	36 months	(+)
	PC ae C34:3	36 months	(+)

### 3.4 Conditional random forest modeling

**Fig. 3:**
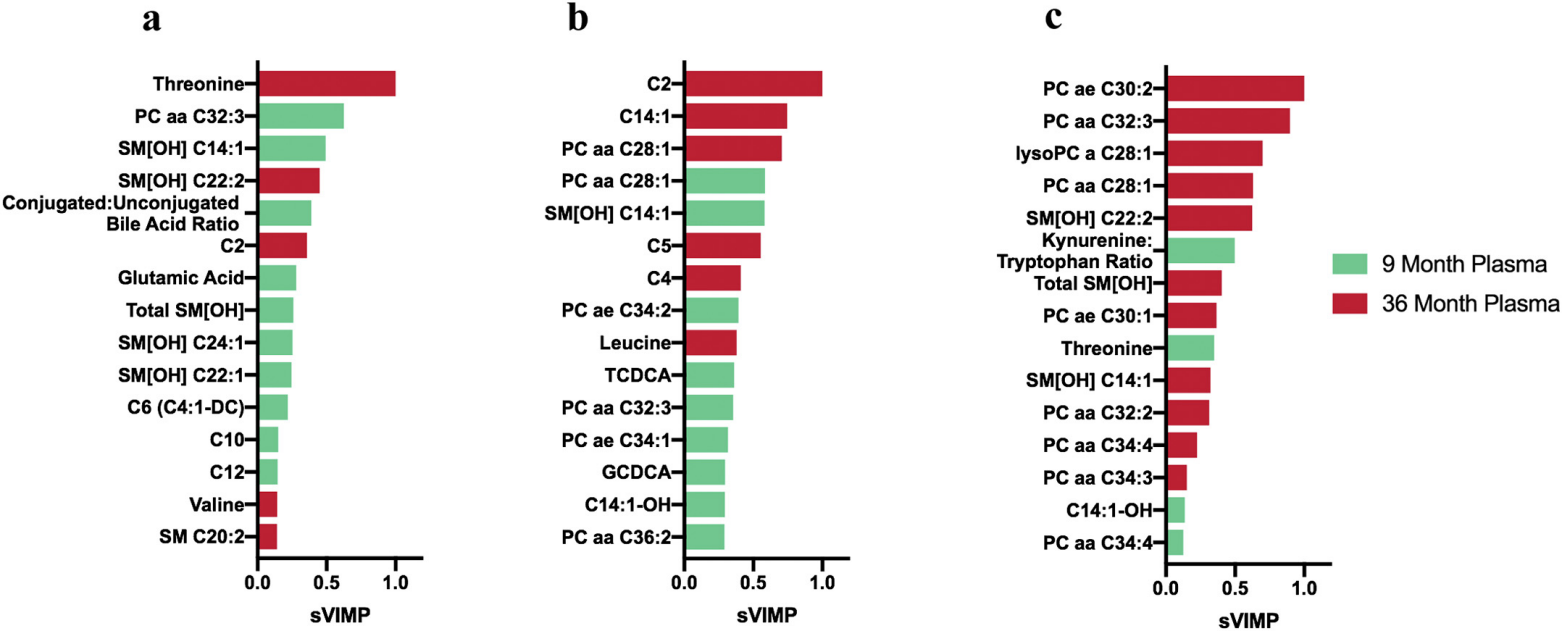
Conditional random forest rankings for four year outcomes: Rankings of the top 15 predictors for (a) HAZ, (b) ∆HAZ, and (c) full-scale IQ score as determined by conditional random forest analysis. Bars represent variable importance scores scaled for each outcome. Nine month metabolites are shown in green while 36 month metabolites are shown in red. (For interpretation of the references to colour in this figure legend, the reader is referred to the web version of this article.)

While correlation and linear regression analysis identified metabolites associated with linear growth and neurocognitive outcomes, a separate method was desired to independently validate these findings. Random forest has become a popular machine learning tool for classification and regression of variables from large biomedical data sets [27,32,33]. Therefore, all measured metabolites from 9 and 36 month plasma were analyzed using a conditional random forest algorithm to select and rank metabolites by their importance to each outcome. Conditional variable importance scores were calculated and scaled within each outcome as a proportion of the highest predictor, which was given a score of 1.0. The top 15 predictors for each outcome as ranked by scaled variable importance (sVIMP) scores are presented in Fig. 3, while the raw and scaled variable importance rankings for all metabolites are reported in Supplemental Table 2.

Overall, there was significant overlap between metabolites identified by our initial analysis and those identified by conditional random forest. Metabolites associated by Pearson's correlation for each outcome made up 100% of the top 15 metabolites selected by conditional random forest for HAZ, 80% for ∆HAZ, and 87% for full-scale IQ score. In addition, metabolites selected by SCAD were also selected by conditional random forest, with 71.4% (5/7) of SCAD-selected HAZ metabolites and 66.7% (4/6) of SCAD-selected full-scale IQ metabolites also being ranked in the top 15 metabolites by conditional random forest. While a smaller proportion (4/13, 30.7%) of SCAD-selected ∆HAZ metabolites were chosen by conditional random forest, 3 of the top 5 random forest predictors were selected by SCAD, providing a higher level of confidence for the most important ∆HAZ predictors. These results suggest that the conditional random forest model largely validates our previous analysis.

The strongest predictors of HAZ outcome were threonine at 36 months sVIMP = 1.0), followed by 9 month PC aa C32:3 (sVIMP = 0.62) and SM[OH] species SM[OH] C14:1 (sVIMP = 0.49) and 36 month SM[OH] C22:2 (sVIMP = 0.45). SM[OH] species were highly ranked predictors of HAZ outcome, making up 6 of the top 15 predictors. For ∆HAZ, the strongest predictors were acylcarnitines C2 (sVIMP = 1.0) and C14:1 (sVIMP = 0.75) at 36 months, followed by PC aa C28:1 from 36 month (sVIMP = 0.71) and 9 month (sVIMP = 0.58) plasma. As observed with correlation analysis, several metabolic pathways were associated with both linear growth outcomes. These include bile acid conjugation (for HAZ, conjugated:unconjugated bile acid ratio, sVIMP = 0.39; for ∆HAZ, bile acids TCDCA, sVIMP = 0.36 and GCDCA, sVIMP = 0.29), acylcarnitines (C2 for both HAZ, sVIMP = 0.36 and ∆HAZ, sVIMP = 1.0), essential amino acids (for HAZ, threonine, sVIMP = 1.0, valine, sVIMP = 0.14, and tryptophan, sVIMP = 0.13; for ∆HAZ, leucine, sVIMP = 0.38), and SM[OH] (5 species for HAZ, SM[OH] C14:1, sVIMP = 0.58, for ∆HAZ). The metabolites associated with neurocognitive development were again distinct from those associated with linear growth. The four top most ranked metabolites for full-scale IQ were 36 month phosphatidylcholines: PC ae C30:2 (sVIMP = 1.0), PC aa C32:3 (sVIMP = 0.90), lysoPC a C28:1 (sVIMP = 0.70), and PC aa C28:1 (sVIMP = 0.63). PC species make up 9 of the top 15 predictors of full-scale IQ. Other metabolic pathways selected by SCAD were also observed in the conditional random forest model, including the kynurenine:tryptophan ratio (sVIMP = 0.50) and threonine (sVIMP = 0.35) from 9 month plasma.

The percentage of variation explained was calculated based on the conditional random forest. This analysis estimates that the conditional random forest model explains 44.7% of HAZ variance, 45.6% of ∆HAZ variance, and 43.1% of full-scale IQ score variance at four years.

## 4 Discussion

The results of this study have implications for the treatment of growth and neurocognitive faltering in undernourished populations. Previous reports suggest that current dietary interventions are insufficient for alleviating growth stunting in children [15,16], potentially due to suboptimal timing or targeting of metabolic pathways. This study has identified key plasma metabolites from both 9 and 36 months that are predictive of four-year growth and neurocognitive outcomes. Of note, several of the most significant 36 month metabolites, including SM[OH] and several PC and acylcarnitine species, were highly correlated between 9 and 36 month timepoints (data not shown). These data indicate that altered levels of these metabolites at early timepoints may predict more pronounced differences later and increase the potential use of these metabolites as biomarkers. Distinct differences were observed between the metabolites associated with growth and those associated with neurocognition, consistent with previous analysis that found different sets of biomarkers to be predictive of these outcomes at two years of age [27]. Differences in metabolites associated with these outcomes suggest that separate metabolic pathways and timings must be targeted for successful intervention.

Growth and neurocognitive outcomes were associated with different classes of metabolites. HAZ and ∆HAZ were represented by all classes measured (bile acids, acylcarnitines, amino acids, phosphatidylcholines, and sphingomyelins) in penalized linear regression and conditional random forest models. The wide range of metabolites associated with growth is consistent with previous studies that found growth outcomes to be associated with multiple factors, including insufficient nutritional intake [10,34], environmental enteric dysfunction (EED) [12,13], and disruption of the intestinal microbiota [35]. Many of the metabolites identified in this study have previously been associated with these disorders [19,22,36]. Poor HAZ and ∆HAZ outcomes were associated with lower concentrations of total essential amino acids in 36 month plasma, particularly threonine, tryptophan, valine, and leucine (Fig. 2A-B), which is likely due to insufficient dietary intake [34]. In addition to being required for protein synthesis, essential amino acids can also inhibit the mTOR pathway, which promotes protein and lipid synthesis [37].Plasma acylcarnitines were also associated with linear growth outcomes, but this association was mixed: short chain acylcarnitines from 36 month plasma were positively associated with growth, while longer chain acylcarnitines from 9 month plasma were negatively associated (Fig. 3A-B). This may be due to differences in function between short and medium-long chain acylcarnitines [38,39] or due to differences in time of measurement. Acylcarnitines are generated as a byproduct of fatty acid oxidation [40], and accumulation of acylcarnitines in plasma is associated with secondary carnitine deficiency, impaired carnitine absorption, or poor diet [41]. EED is known to promote impaired absorption due to tissue damage and villi stunting [42], and acylcarnitine accumulation has been associated with elevated gut permeability [22]. Several other metabolites link growth to impaired absorption through EED, including threonine, a major component of mucus glycoproteins that line the intestines [43] and play a critical role in maintaining intestinal barrier function [44,45] as well as glutamic acid, which was elevated in children with poor growth outcomes (Table 3) and has previously been associated with intestinal permeability [20]. Finally, high levels of bile acid conjugation were associated with poor HAZ and ∆HAZ outcomes (Table 3), which suggests a role for the intestinal microbiota. While bile acid conjugation is a host process that occurs in the liver, deconjugation is performed by intestinal microbes through the activity of bile salt hydrolases (BSH) [46]. BSH-expressing bacteria in the gut have been linked to proper lipid metabolism by influencing host gene expression [47], so disruption of these microbial communities may lead to impaired growth.

While many of these metabolites have previously been associated with growth outcomes, this study found a significant role for both SM[OH] and PC species, which were the most abundant metabolites associated with both HAZ and ∆HAZ (Table 2). PCs are the most abundant phospholipid in mammalian cell membranes [48] and are critical for cell membrane structure and physiology as well as optimal bone growth and development [49,50]. Both total SM[OH] and individual SM[OH] species were strongly associated with growth outcomes by all methods of analysis. An association between higher serum concentrations of some SM[OH] species and improved HAZ was observed in a child cohort in rural Malawi [20], but the authors did not speculate on this finding. The mechanism by which SM[OH] contributes to stunting is not well characterized. No significant associations were observed between four year outcomes and non-hydroxylated sphingomyelins, suggesting that this phenotype is specific to the hydroxylated form of the sphingolipid. Synthesis of hydroxylated sphingomyelin is identical to synthesis of non-hydroxylated sphingomyelin except for the addition of a hydroxyl group to the fatty acid chain by fatty acid-2-hydroxylase (FA2H) before attachment to the ceramide backbone [51]. Most literature regarding sphingolipid hydroxylation pertains to effects on neurocognition, as hydroxyl-sphingolipids are enriched in myelin sheaths [52] and mutation of FA2H has been associated with neurodegeneration [[53], [54], [55]]. These results may explain the observed associations between SM[OH] and neurocognitive outcome (Table 2). FA2H has also been implicated in cAMP signaling [56] and glucose uptake in adipocytes [57], which may explain the growth phenotypes observed.

While metabolic changes have previously been associated with linear growth outcomes, the literature regarding metabolic associations with neurocognition is lacking. Therefore, the current study represents an important overview of metabolic trends that are associated with neurocognitive scoring in an undernourished child cohort. Metabolites associated with full-scale IQ score were disproportionately PC species, which make up 65% of all metabolites associated by Pearson correlation (Table 2) and nine of the top 15 predictors of full-scale IQ by conditional random forest (Fig. 3). Decreased plasma PC concentration has been linked previously to decreased cognitive outcomes in Alzheimer's Disease [58,59], and PC components such as choline [60,61] and specific fatty acids [62,63] have been associated with neurocognitive development. The mechanism by which PCs are associated with neurocognitive development has not been elucidated, but may be due to crosstalk with inflammation. PC is important for maintaining barrier function in the gut [64], and increased intestinal PCs have been associated with decreased intestinal inflammation [[65], [66], [67]]. While much of this data is based on intestinal PCs, plasma PCs may have similar anti-inflammatory effects, which could explain how decreased plasma PC is deleterious to neurocognitive development. While the current study observed an association between phosphatidylcholine species and full-scale IQ score, analysis of lipids was semi-quantitative and lacked the resolution necessary to distinguish which specific fatty acid chains make up each PC species identified (Supplemental Table 3). Further studies are necessary to investigate whether individual fatty acid components are associated with proper neurocognition in undernourished children.

Other metabolites identified in this study emphasize the impact of early inflammation on neurocognition. Non-PC metabolites associated with neurocognitive outcomes include threonine, which was associated with improved outcomes, as well as the biogenic amine kynurenine and the kynurenine:tryptophan ratio (Kyn:Trp), which were associated with poor outcomes (Table 3, Fig. 3). The importance of threonine to proper gut integrity as a mechanism to prevent inflammation has previously been described. Elevated kynurenine and Kyn:Trp ratio are markers of increased indoleamine 2,3-dioxygenase 1 (IDO1) activity, an enzyme that converts tryptophan to kynurenine in response to inflammatory signals [68]. High Kyn:Trp ratio has previously been associated with stunting in other cohorts [69] as well as HIV-associated neurocognitive disorders [70]. This pathway has also been implicated in regulation of inflammation in the central nervous system [71], providing a mechanistic link between gut inflammation and neural development.

In addition to differences in the metabolites associated with each outcome, there were also differences in the times at which these metabolites were measured. Linear growth outcomes were more likely to be associated with 9 month metabolites, which made up 61.8% of associated metabolites for HAZ and 51.4% for ∆HAZ by Pearson correlation analysis (Table 2). Previous studies have shown that maternal and birth anthropometry influence linear growth throughout childhood [[72], [73], [74]]. In contrast, full-scale IQ score was largely associated with 36 month metabolites, which made up 85.3% of metabolites associated by Pearson correlation (Table 2), 64.7% associated by SCAD (Table 3), and 73% of the top 15 metabolites associated by conditional random forest (Fig. 3C). Neurocognition in the PROVIDE cohort has previously been assessed at earlier timepoints using the Bayley Scales of Infant and Toddler Development, Third Edition (Bayley-III) [[25], [26], [27]]. When metabolic associations with Bayley-III scores at 18 and 24 months were explored, PC species from 9 month plasma were not significantly associated, but associations with several 24 month PC species were observed (Supplemental Fig. 2). All associations with 24 month plasma metabolites were not assessed because this data was only collected on a smaller subset (75/130) of this cohort, but these results suggest that the association between PCs and neurocognition may represent a later (2+ years post-birth) biological phenomenon. As this is later in childhood and children are no longer exclusively breastfeeding at this point, this may be driven by environmental stressors rather than maternal or birth factors. Childhood neurodevelopment has been previously linked to environmental stressors that may be the predominant influences on metabolic status later in childhood, including the intensity of disease burden [75] or inflammation [76,77].

In this study, plasma metabolites were analyzed for associations with linear growth and neurocognitive development at four years using two independent methods. The sets of metabolites selected using these methods were largely consistent, with over 80% of the top 15 ranked metabolites by conditional random forest associated with outcomes by Pearson correlation. The similarity of metabolite sets identified by these methods reinforces that the metabolites selected represent the strongest predictors of growth and neurocognitive development in this population. Many of the associations observed in the current study are also consistent with previous findings from other groups, including associations for essential amino acids [20,23], bile acid conjugation [28,36], and acylcarnitines [22] with EED and growth outcomes. While the large number of metabolites assayed by the p180 kit resulted in few metabolites meeting a false discovery threshold for significance, the consistency of these results with previous findings instills confidence that these associations are not just artifacts of the large number of metabolites analyzed.

Important questions remain as to the mechanism by which these metabolites are elevated or decreased in the context of undernutrition. While dietary differences may contribute to impaired linear growth or neurocognitive outcomes, members of this cohort live within the same community and likely have similar diets. Plasma metabolites may instead be reflective of environmental stressors such as chronic inflammation or differences in the intestinal microbiota, as changes in microbiome composition can profoundly affect growth [[78], [79], [80]] and neurodevelopmental [81,82] outcomes. Work is currently ongoing to investigate metagenomic data within this cohort for associations with four year outcomes, which may provide a mechanistic link for some of the metabolic associations observed in this study.

While distinct differences were observed between growth and neurocognitive outcomes, there were some similar pathways that may serve as effective targets for intervention. Indicators of inflammation and malabsorption associated with EED were observed at 9 months with both growth (conjugated bile acids, long chain acylcarnitines) and neurocognition (threonine, Kyn:Trp), suggesting that early interventions targeting EED may be effective for both outcomes. In addition, lipid metabolites were associated with both outcomes (Table 2), including several individual SM[OH] and PC species (PC aa C32:3, PC aa C28:1) (Fig. 3). These data indicate that lipid metabolism may be a potential pathway of interest for intervention. There is some evidence that lipid-based interventions may be effective for the treatment of acute malnutrition [83]. In addition, a recent trial in Ecuador found that dietary supplementation with eggs resulted in significant HAZ recovery in children [84], potentially by boosting plasma levels of choline [85]. Future work is necessary to see if this is consistent in other populations, but lipid biosynthesis may represent a novel target for intervention.

The following are the supplementary data related to this article.

Supplementary Figure 1 Supplementary Figure 1

Supplementary Figure 2 Supplementary Figure 2

Supplementary Table 1 List of all metabolites associated with four year outcomes with P and r values Supplementary Table 1

Supplementary Table 2 Raw and Scaled Variable Importance Scores for All Metabolites Analyzed by Conditional Random Forest Supplementary Table 2

Supplementary Table 3 List of Isobaric and Isomeric Lipid Species Measured by the AbsoluteIDQ p180 Kit® (Adapted from https://www.biocrates.com/images/List-of-Isobaric-and-Isomeric-Lipid-Species_v1_2018.pdf) Supplementary Table 3

Lists of metabolites analyzed and all associations with outcome Image 1
