# Supplementary material for: Childhood growth and neurocognition are associated with distinct sets of metabolites
Source: eBioMedicine. 2019 May 25;44:597–606. doi: 10.1016/j.ebiom.2019.05.043 (PMC6604877; doi:10.1016/j.ebiom.2019.05.043)
Supplement: Supplementary Figure 1 [file mmc1.docx]

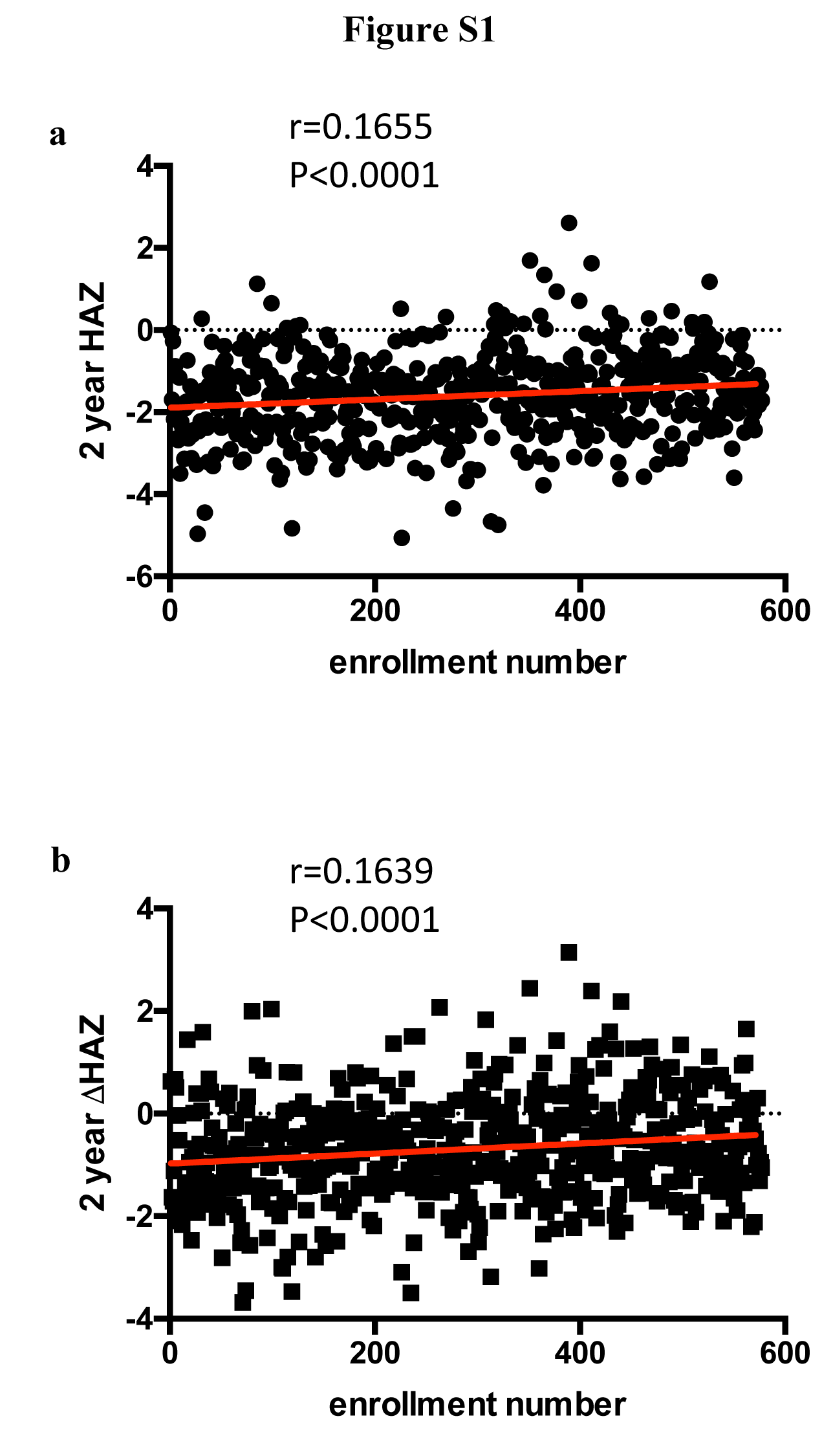


**Supplementary Figure 1: HAZ and ∆HAZ Achievement over the Course of PROVIDE Enrollment:** Two year (a) HAZ and (b) ∆HAZ measurements were examined for children from the full PROVIDE cohort over the 1.5 year course of enrollment, with enrollment number used as a proxy for enrollment over time. Pearson correlation r and p values are reported for each plot. Both HAZ and ∆HAZ significantly increased over the course of enrollment.


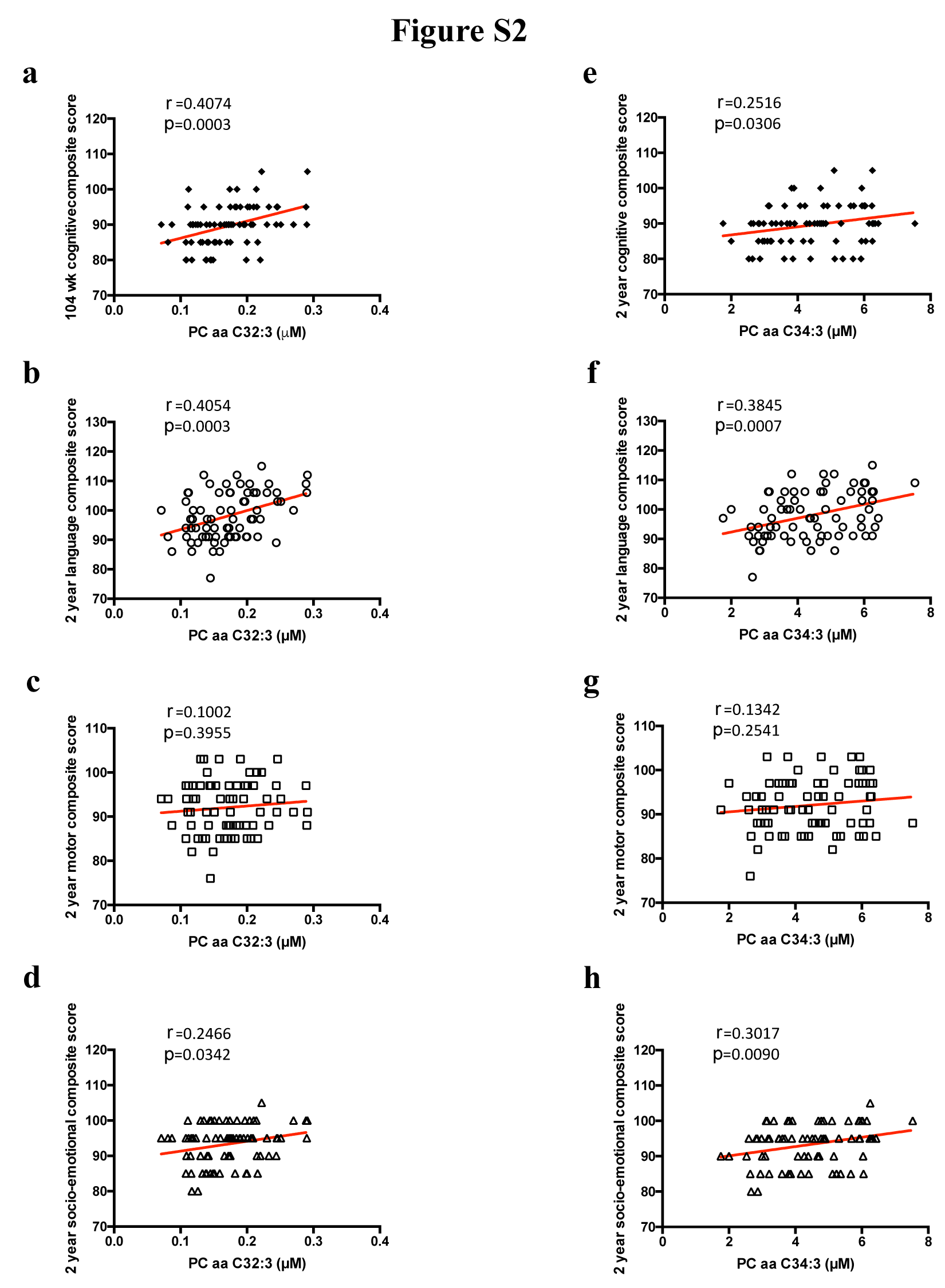


**Supplemental Figure 2: Association of Two Year Neurocognitive Scoring (Bayley-III) and Two Year PC Species:** Correlations between two of several two year PC metabolites associated with Bayley-III score at two years: (a-d) PC aa C32:3 and (e-h) PC aa C34:3. The Bayley-III is divided into four composite scores: (a,e) Cognition, (b,f) Language, (c,g) Motor, and (d,h) Socio-emotional. Pearson correlation r and p values are reported for each plot.
